# Prevalence of generalized joint hypermobility in children with anxiety disorders

**DOI:** 10.1186/s12891-020-03377-0

**Published:** 2020-06-02

**Authors:** Vadood Javadi Parvaneh, Shadialsadat Modaress, Ghazal Zahed, Khosro Rahmani, Reza Shiari

**Affiliations:** 1 Department of pediatric rheumatology, Mofid Children’s Hospital, Shahid Beheshti University of Medical Sciences, Tehran, Iran; 2 Child and Adolescent Psychiatry department, Mofid Children’s Hospital, Shahid Beheshti University of Medical Sciences, Shariati Ave, Hosseinieh Ershad, Tehran, Iran

**Keywords:** Anxiety disorders, Hypermobility, Beighton, Shiari-Javadi, Children

## Abstract

**Background:**

Concerning the high prevalence of anxiety disorders and joint hypermobility in children and the lack of related studies in this age group, we aimed to assess the association of hypermobility with anxiety disorders in children.

**Methods:**

In this case-control study, 93 children ages 8–15 years with anxiety disorders referring to the Child and Adolescent Psychiatry Clinic of Mofid Children’s Hospital, Tehran, Iran, during 2018, were enrolled. The control group consisted of 100 age and sex-matched children without anxiety disorders. Anxiety was evaluated using the Spence Children Anxiety Scale (SCAS). The diagnosis of generalized joint hypermobility was done based on Beighton and Shiari-Javadi criteria.

**Results:**

Based on Beighton’s diagnostic criteria 52.7% of the children in the case group and 16% of the children in the control group had generalized joint hypermobility. Moreover, based on Shiari-Javadi criteria, 49.5 and 13% of the children in the case and control groups had generalized joint hypermobility, respectively. Moreover, the internal correlation between the two criteria was 0.91 showing almost complete compatibility between the two (*P* <  0.001). Age was a risk factor that could predict hypermobility in these children. Other variables such as sex, severity, and type of anxiety disorders, and ADHD, were not predictors of hypermobility syndrome.

**Conclusion:**

The prevalence of hypermobility was three times higher in children with anxiety disorders and only age was a predictor for the possibility to suffer from generalized joint hypermobility in these children.

## Background

The term joint hypermobility was first introduced to the medical dictionary in the late nineteenth century to define Marfan and Ehlers-Danlos syndromes [1]. Although joint hypermobility is characteristic of several collagen disorders such as Marfan, Ehlers-Danlos syndromes and osteogenesis imperfecta, generalized hypermobility can also be seen in a relatively high percentage of healthy individuals [2, 3]. The prevalence of hypermobility ranges from 7 to 38.5% depending on age, sex, and race [4]. Joint hypermobility-related disorders are on a wide spectrum, ranging from asymptomatic generalized hypermobility to symptomatic hypermobility which includes hypermobility spectrum disorders (HSDs) and hypermobile Ehlers-Danlos syndrome (hEDS). HSDs are relatively common in childhood. All patients with generalized joint hypermobility, which have musculoskeletal complaints or comorbidities related to hypermobility, are diagnosed as having hypermobility spectrum disorders if they do not fulfill hypermobile Ehlers-Danlos syndrome and other hereditary connective tissue disorders criteria [5–8]. Recent studies show that hypermobility spectrum disorder is not restricted to the motor system or joints and could be associated with comorbidities such as mitral valve prolapse, visceral symptoms, and psychological problems [9, 10].

So far, many studies have been done on the psychological manifestations of hypermobility. In a systematic review of 35 studies on the psychological manifestations of joint hypermobility, joint hypermobility syndrome, and Ehlers-Danlos syndrome, of which 21 assessed the association between anxiety disorder and joint hypermobility. Seventeen articles showed a statistically significant association between anxiety disorders and joint hypermobility, while 4 did not find such a relationship. It should be stated that all these 21 studies were done on the adult population and to the best of our knowledge no related study has been so far done on children [11]. In another study comparing the grey matter volume in adults with anxiety disorders and healthy individuals, the researchers found a significant relationship between the size of the amygdala and the hypermobility score in these patients, while no such relationship was reported in non-anxious adults [12]. The amygdala contributes to emotional processing such as anxiety, fear, and sadness. It is highly responsive to emotional and stressful events. Consistently, hypermobility syndrome has been identified as a risk factor predicting anxiety disorders in adults over 60 years [13].

The prevalence of mental disorders in children and adolescents that moderately interferes with their performance has been reported to be around 20%, among which anxiety disorder is the most prevalent (13%) [14]. The coexistence of psychological problems and physical illnesses such as neuromuscular diseases deteriorate the patient’s condition and lead to an unsuitable therapeutic response to current treatments. This is because most physical illnesses debilitate children which would in turn increase anxiety and its types such as generalized anxiety. Moreover, the parents’ concerns would lead to increased tension and anxiety in the family which intensifies anxiety disorder [15].

## Methods

### Study design and participants

In this case-control study, 93 children ages 8–15 years with anxiety disorders referred to the Child and Adolescent Psychiatry Clinic of Mofid Children’s Hospital, Tehran, Iran, during 2018, were enrolled consecutively. The control group consisted of 100 age and sex-matched children who visited in emergency and surgery clinics without any chronic or underlying diseases and without anxiety disorders. Anxiety was evaluated using the Spence Children Anxiety Scale (SCAS). This scale was developed by Spence in 1997 to assess anxiety in 8–15-year-old children based on the DSM-IV categorization. It has two versions: the child version with 45 items and the parent version with 38 items, evaluating six domains. It is scored on a four-item Likert scale from zero to three. The six subscales of the SCAS are as follows: separation anxiety, social phobia, obsessive-compulsive disorder, panic/agoraphobia, generalized anxiety, and specific phobia [16]. The validity of the scale for the Iranian population has been previously confirmed with a Cronbach’s alpha of 0.86 [17]. The criteria for confirming anxiety are as follows: total score of > 40 in boys and > 50 in girls aged 8–11 years, and total score of > 33 in boys and > 40 in girls aged 12–15 years. Scores of 0–44 show mild anxiety, 44–88 moderate anxiety, and over 88 depict severe anxiety in children.

We have previously proposed the new specific diagnostic criteria for children (Shiari-Javadi) and compare it with Beighton criteria in most our researches about the hypermobility. In this study, the diagnosis of generalized joint hypermobility was done by a pediatric rheumatologist based on Beighton [18] and Shiari-Javadi criteria [19]. Beighton’s scores range from zero to nine and scores higher than six show hypermobility [18, 20], while according to the Shiari-Javadi criteria, scores range from zero to eight, and scores higher than six show hypermobility of the joints. (Table 1).

**Table 1:** Maneuvers and scores of Shiari-Javadi Criteria for generalized joint hypermobility

Numbers	Maneuvers	score
1	Passive back hyperextension > 30°	1
2	Bilateral passive lateral neck rotation in which nose crosses	1
	Imaginary line down the frontal appendage of acromio-clavicular joint	
3	Bilateral passive MCPS hyperextension > 90°	1
4	Elbows hyperextension ≥10°	1
5	Vertical shoulders hyperextension so that elbows touch together from behind	1
6	Passive hips abduction so that the angle between them > 120°	1
7	Passive knees hyperextension > 10°	1
8	Thumbs hyperextension so that thumbs touch volar aspect of forearm	1
Total Scores		8

### Statistical analysis

Data were analyzed using SPSS software, version 16. Mean and standard deviations, as well as frequencies, were calculated as appropriated. Chi-square and t-tests were used to assess the association between variables and between-group differences. ROC curve analysis was done to assess the diagnostic specificity and sensitivity of the Shiari-Javadi criteria compared with Beighton’s criteria. Binary logistic regression was used to identify risk factors of hypermobility syndrome in children with an anxiety disorder. We evaluated the association of variants including age, gender, anxiety severity, anxiety type, and ADHD with hypermobility. Other factors such as environmental or genetic factors were not in the aims of the study. The Nagelkerke R^2^ values in logistic regression models for Beighton and Shiari-javadi criterias were 0.23 and 0.24 respectively. *P* <  0.05 was considered statistically significant.

## Results

In the case group, 93 children with anxiety disorders were assessed and compared with 100 children without an anxiety disorder. There was no statistically significant difference between both groups concerning age and sex (Table 2). Based on Beighton’s diagnostic criteria 52.7% of the children in the case group and 16% of the children in the control group had generalized joint hypermobility. Moreover, based on Shiari-Javadi criteria, 49.5 and 13% of the children in the case and control groups had generalized joint hypermobility, respectively. A significant difference was found between the rate of generalized joint hypermobility based on the two criteria in the case and control groups (Kapp = 0.834, *P* <  0.001, Table 2). Moreover, the internal correlation between the two criteria was 0.91 showing almost complete compatibility between the two (95%CI: 0.88–0.93, *P* <  0.001). Sensitivity, specificity, positive and negative predictive values, effectiveness, and area under ROC curve were 96.9, 84.6, 92.5, 93.2, 92.7, and 0.907%, respectively.

**Table 2:** Characteristic data and diagnosis of joint hypermobility syndrome in case and control’s children

	Case group *n* = 93	Control group *n* = 100	*P*-value
Sex^a^ Male	59 (63.4)	59 (59)	0.55
Age^b^ Year	10.3 ± 2.1	10.6 ± 2.0	0.14
Hypermobility syndrome			
Beighton^a^	49 (52.7)	16 (16)	< 0.001
Shiari-Javadi^a^	46 (49.5)	13 (13)	< 0.001

a = n(%), b = Mean ± SD

With respect to the severity of anxiety disorders in the case group, 37 (39.8%) children had mild anxiety and 56 (60.2%) had moderate anxiety. With respect to type, 63 (67.7%) children had separation anxiety, 30 (32.3%) had social phobia, 45 (48.4%) had obsessive-compulsive disorder, 40 (43%) had panic/agoraphobia, 68 (73.1%) had specific anxiety, and 85 (91.4%) had generalized anxiety. Moreover, in clinical examination, other psychiatric disorders were also diagnosed such as Attention Deficit/Hyperactivity Disorder (ADHD, *n* = 30, 32.3%), mood disorders (*n* = 9, 9.7%), Stereotypic Movement Disorders (*n* = 3, 3.2%), school refusal (*n* = 2, 2.2%), motor tic (*n* = 2, 2.2%), somatic symptom disorder (*n* = 1, 1.1%), learning disability (*n* = 1, 1.1%), and functional constipation (*n* = 1, 1.1%).

We found that based on Beighton’s criteria, in children with anxiety disorders, those who were younger had a higher risk of suffering from generalized joint hypermobility (Table 3). As shown in Table 2, other variables such as sex, severity and type of anxiety disorders, and ADHD, were not predictors of generalized joint hypermobility. Repeating the evaluations using the Shiari-Javadi criteria yielded similar results to those of Beighton’s criteria (Table 4).

**Table 3:** Risk factors of JHS in children with AD based onBeighton criteria

	JHS n = 49	Non JHS n = 44	Sig	OR	95% CI	
					Lower	Upper
Sex^a^ male	28 (57.1)	31 (70.5)	0.07	0.39	0.14	1.09
Age^b^ year	9.69 ± 1.93	10.89 ± 2.13	0.02	0.75	0.59	0.95
AD Severity						
Mild^a^	22 (44.9)	15 (34.1)	0.06	0.27	0.06	1.08
Moderate^a^	27 (55.1)	29 (65.9)				
AD Type						
Separation anxiety	34 (69.4)	29 (65.9)	0.25	2.20	0.57	8.43
Social phobia	17 (34.7)	13 (29.5)	0.22	2.05	0.65	6.50
OCD	22 (44.9)	23 (52.3)	0.92	0.95	0.36	2.48
Panic/agoraphobia	19 (38.8)	21 (47.7)	0.77	0.82	0.23	2.95
Specific phobia	36 (73.5)	32 (72.7)	0.70	0.79	0.25	2.55
Generalized anxiety	44 (89.8)	41 (93.2)	0.88	1.14	0.18	7.12
ADHD	12 (24.5)	18 (40.9)	0.05	0.36	0.12	1.03

a = n(%), b = Mean ± SD. Abbreviations: *JHS* Joint Hypermobility Syndrome, *AD* Anxiety Disorders, *OCD* Obsessive-Compulsive Disorder

**Table 4:** Risk factors of JHS in children with AD based on Shiari-Javadi criteria

	JHS n = 46	Non JHS n = 47	Sig	OR	95% CI	
					Lower	Upper
Sex^a^ male	26 (56.5)	33 (70.2)	0.06	0.38	0.13	1.04
Age^b^ year	9.55 ± 1.92	10.95 ± 2.06	0.01	0.72	0.56	0.92
AD Severity						
Mild^a^	20 (43.5)	17 (36.2)	0.06	0.26	0.06	1.10
Moderate^a^	26 (56.5)	30 (63.8)				
AD Type						
Separation anxiety	34 (73.9)	29 (61.7)	0.09	3.24	0.82	12.82
Social phobia	16 (34.8)	14 (29.8)	0.32	1.77	0.57	5.53
OCD	21 (45.7)	24 (51.1)	0.97	0.98	0.37	2.57
Panic/agrophobia	18 (39.1)	22 (46.8)	0.70	0.78	0.21	2.82
Specific phobia	35 (76.1)	33 (70.2)	0.86	0.90	0.27	2.94
Generalized anxiety	41 (89.1)	44 (93.6)	0.73	1.38	0.21	8.76
ADHD	13 (28.3)	17 (36.2)	0.29	0.57	0.20	1.60

## Discussion

Hypermobility is a tissue-related clinical condition that is usually hereditary and more prevalent in women [21]. In this study, we used two diagnostic criteria for diagnosing hypermobility of the joints. We found that the two criteria were highly compatible with each other. On the basis of the international consensus statement on the Beighton score 2017, Beighton criteria are the standard criteria with acceptable inter-rater reliability in clinical practice [18]. However, it has been proposed for all age groups and it is not specific for children. Furthermore, it has some limitations in pediatric group such as: a) performing the forward bending maneuver in young children is difficult and challenging, b) hypermobility of cervical and total spinal column has been ignored, while this is important in children with hypermobility, c) large joints such as shoulders and hips have not been considered or underscored. For children, one cut-off point, varying from 5 to7 was used in Beighton score (5/9, 6/9, and 7/9). So, we used cut-off point>6/9 for both Beighton and Shiari-Javadi criteria.

Moreover, we found a significant association between generalized joint hypermobility and anxiety disorders in children, so that this phenotype was three times more prevalent in children with anxiety disorders. This finding is consistent with several previous studies [13, 16, 18–24]. The mentioned studies were all done on the adult population and we found such association in children as well. The most prevalent anxiety disorders were generalized anxiety (61.4%), specific phobia (73.1%), and separation anxiety (67.7%), respectively. Anxiety is associated with pain-related fear in hypermobile kids. In profoundly anxious hypermobile youths, tension rather than pain gives off an impression of being related to limitations in social and physical working. There might be a common pathway for hypermobility and anxiety. Moreover, in certain inquires about the seriousness of joint hypermobility and anxiety even showed up positively related [25].

We found that more than half the children with anxiety disorders along with hypermobility were boys, which is inconsistent with other studies [26, 27]. This is while based on regression analysis in our study sex was not a predicting factor for hypermobility in children with anxiety disorders. Age evaluations in children with anxiety disorders showed that the mean age in children with generalized joint hypermobility was significantly lower than those without hypermobility (9.69 vs 10.89 years based on Beighton’s criteria and 9.55 vs 10.95 based on Shiari-Javadi’s criteria). Age could significantly predict hypermobility in children with anxiety disorders in our study (OR = 0.75 CI 95% = 0.59–0.95 based on Beighton’s criteria and OR = 0.72 CI 95% = 0.56–0.9 based on Shiari-Javadi’s criteria), which is consistent with another study [28].

The severity of anxiety disorders was moderate in our sample (60.2%) and this variable could not significantly predict hypermobility in children with anxiety disorders. Type of anxiety disorder has a similar condition with respect to predicting hypermobility. This is while Bulbena and colleagues found that the prevalence of panic/agoraphobia, social phobia, and simple phobia was higher in people with hypermobility [22]. This inconsistency could be attributed to the different protocols of the study; in the mentioned study the case group comprised 29 people with hypermobility and the control group comprised 108 people without it, while in our study the case group consisted of children with anxiety disorders and the control group comprised children without anxiety disorders.

We also found that comorbidity of ADHD with an anxiety disorder was not a significant predictor of hypermobility, which is inconsistent with two other studies showing a higher prevalence of hypermobility in children with ADHD (15, 29). In the mentioned studies ADHD was considered as the main disorder whose relationship with hypermobility was assessed while we considered ADHD alongside anxiety disorders.

## Conclusion

The prevalence of hypermobility was higher in children with anxiety disorders. Age was a risk factor that could predict hypermobility in these children. Other variables such as sex, severity, and type of anxiety disorders, and ADHD, were not predictors of generalized joint hypermobility.

Although generalized joint hypermobility is a physiologic musculoskeletal variant in children without complaints, this study in children and similar findings in adults proposed that in approach to anxiety or hypermobility, the clinician should be alert about the association of these problems. In a child with an anxiety disorder, the practitioner should focus on somatic complaints in history taking and on generalized joint hypermobility in the physical examination. This matter may be a clue for the diagnosis of joint hypermobility syndrome. This finding (due to having pain) can be a reason for induction or intensifying anxiety in a child. This association alertness may help the physician in disease management, so that with intervention to manage each of these situations the result may affect others.

## Data Availability

If requested (please contact vadoodj@gmail.com).

## Abbreviations

SCAS: Spence children anxiety scale
ROC curve: Receiver operating characteristic curve
ADHD: Attention Deficit/Hyperactivity Disorder
OR: Odd's ratio
CI: Confidence interval
